# Collision Tumor of Gallbladder Carcinosarcoma and Intrahepatic Cholangiocarcinoma: A Rare Case Report

**DOI:** 10.70352/scrj.cr.24-0160

**Published:** 2025-05-08

**Authors:** Zen Naito, Takehiro Noji, Kimitaka Tanaka, Yoshitsugu Nakanishi, Masahito Nakajima, Tomoko Mitsuhashi, Satoshi Hirano

**Affiliations:** 1 Department of Gastroenterological Surgery II, Faculty of Medicine, Hokkaido University, Sapporo, Hokkaido, Japan; 2 Hokkaido Nursing Division, Faculty of Health Sciences, Hokkaido University, Sapporo, Hokkaido, Japan; 3 Aiiku Hospital, Sapporo, Hokkaido, Japan; 4 Surgical Pathology, Hokkaido University, Sapporo, Hokkaido, Japan

**Keywords:** collision tumor, gallbladder carcinosarcoma, adenosquamous carcinoma, intrahepatic cholangiocarcinoma

## Abstract

**INTRODUCTION:**

Gallbladder carcinosarcoma is extremely rare, with fewer than 100 cases reported from its first description in 1907 until 2022. Collision carcinoma is a type of synchronous carcinoma in which 2 independently arising tumors come into contact or partially invade each other.

**CASE PRESENTATION:**

A man in his 80s was referred to our hospital with the primary complaints of weight loss and decreased appetite. Contrast-enhanced computed tomography revealed a large lobular tumor with heterogeneous enhancement, measuring 66 mm in maximum diameter, located in the fundus of the gallbladder. The mass showed clear signs of liver invasion, raising immediate concerns of malignancy. Magnetic resonance imaging provided additional crucial details. The lesion exhibited low signal intensity on T1-weighted images and high signal intensity on T2-weighted images. Notably, diffusion-weighted imaging demonstrated restricted diffusion, a characteristic often associated with malignant processes. These findings strongly suggested gallbladder cancer with liver invasion. The patient underwent cholecystectomy and hepatectomy involving segments 4, 5, and 8 of the liver. A subsequent pathological examination revealed a complex and unusual tumor composition. The hepatic lesion showed nests of varying sizes with a medullary growth pattern, which is characteristic of intrahepatic cholangiocarcinoma. In contrast, the gallbladder lesion displayed features of adenosquamous carcinoma with a partial sarcomatoid morphology, indicative of gallbladder carcinosarcoma. Intriguingly, the interface between these 2 distinct tumor types exhibited unique characteristics. In some areas, normal hepatocytes were interspersed between the 2 types of tumor cells. Other regions demonstrated an invasive tendency of tumor cells towards each other. This unusual pattern led to the diagnosis of a collision tumor, a rare occurrence in which 2 independent primary malignancies coexist in the same organ or site.

**CONCLUSIONS:**

This was an extremely rare case of collision carcinoma involving both intrahepatic cholangiocarcinoma and gallbladder carcinosarcoma. The unique pathological findings and rarity of this tumor combination make this case particularly noteworthy. We present this case to contribute to the limited literature on such rare tumors, aiming to facilitate a better understanding and management of similar cases in the future.

## Abbreviations

CT
: computed tomography
DWI
: diffusion-weighted imaging
HE
: hematoxylin and eosin
MRI
: magnetic resonance imaging

## INTRODUCTION

Gallbladder carcinosarcoma constitutes less than 1% of all gallbladder cancers^1)^ and is extremely rare, with fewer than 100 cases reported from its first description by Landsteiner in 1907 until 2022.^2)^ Collision carcinoma is a type of synchronous carcinoma in which 2 independently arising tumors come into contact or partially invade each other.^3)^

Herein, we report an extremely rare case of a collision tumor involving gallbladder carcinosarcoma and intrahepatic cholangiocarcinoma.

## CASE REPORT

A man in his 80s was admitted to our hospital complaining of weight and appetite loss during a follow-up for myelodysplastic syndrome. The CA19-9 level on admission was 545 U/mL, and the patient had no other significant abnormalities.

**Fig. 1:**
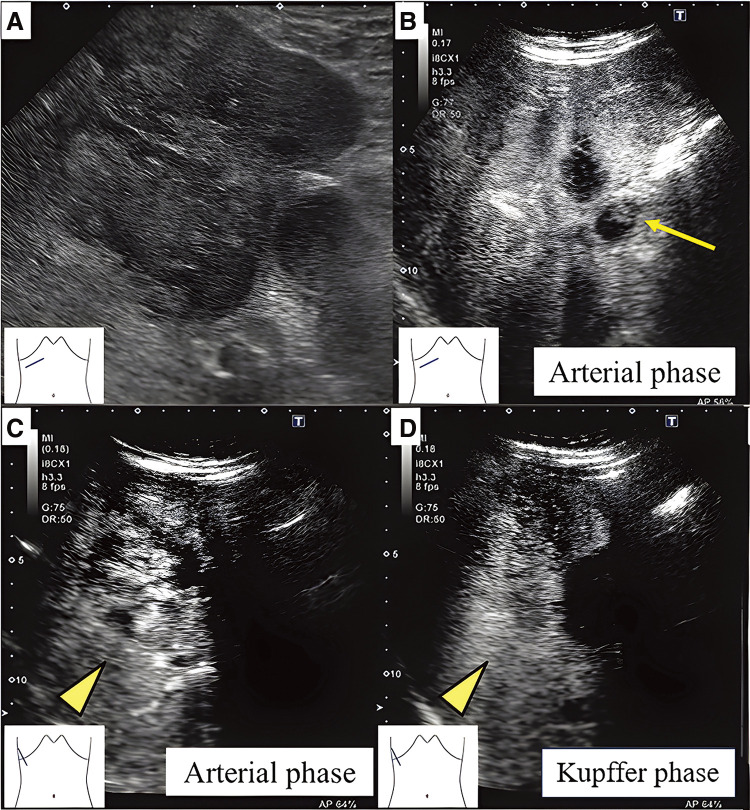
Tumorous lesion extending from the gallbladder to the liver segment 8 (**A**). The enhanced echo image of the gallbladder fundus showing a gallbladder tumor (**B**: arrow). Multinodular internal enhancement during the arterial phase and a reduction in this enhancement during the portal phase (**C** and **D**: arrowhead).

Abdominal and contrast-enhanced ultrasonography using Sonazoid (GE Healthcare, Chicago, IL, USA) revealed an 80 × 70 × 66 mm heterogeneous tumor with cystic areas extending from the fundus of the gallbladder to the liver, multiple nodular enhancements in the arterial phase, reduced enhancement in the Kupffer phase, and non-enhanced areas in both phases. The hepatic lesion was in contact with the middle hepatic vein, suggesting invasion (**Fig. 1**).

**Fig. 2:**
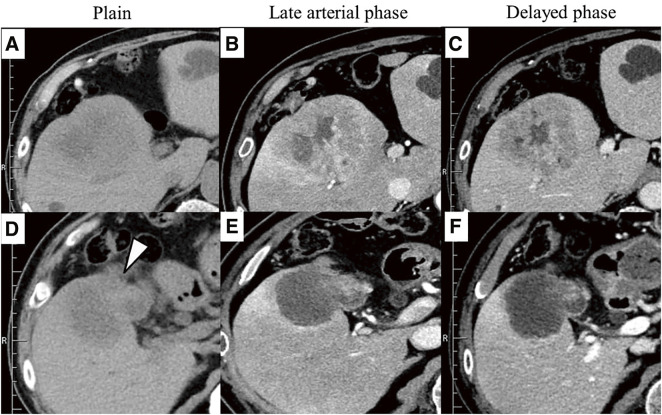
A lobulated tumor with lower density than normal liver tissue (**A**), showing a gradual and heterogeneous ring enhancement toward the interior (**B** and **C**). A tumorous lesion extending from the gallbladder (**D**: arrowhead), with enhancement observed at the fundus, but no liver-enhanced lesions that are part of the tumor (**E**, **F**).

Computed tomography (CT) revealed an 80 × 70 mm tumor in the gallbladder fundus invading segment 8 of the liver. The hepatic lesion was heterogeneous, with ring enhancement in the late arterial phase, and gradual and prolonged enhancement in the equilibrium phase. The hepatic and gallbladder lesions showed identical enhancement patterns. No apparent lymph node or distant metastases were observed (**Fig. 2**).

**Fig. 3:**
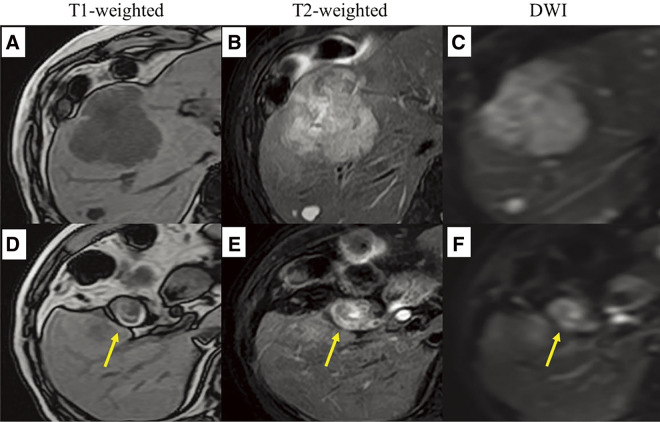
The liver lesion showed low intensity on T1-weighted images, high intensity on T2-weighted images, and diffusion restriction on DWI (**A**–**C**). The gallbladder lesion showed similar findings (**D–F**: arrow). DWI, diffusion-weighted image

Magnetic resonance imaging (MRI) revealed a lesion in the gallbladder fundus with low intensity on T1-weighted images, high intensity on T2-weighted images, and restricted diffusion on diffusion-weighted imaging (DWI). The hepatic lesions exhibited mixed low and high intensities on T2-weighted images, low intensity on T1-weighted images, and restricted diffusion on DWI. No other metastatic lesions were observed (**Fig. 3**).

**Fig. 4:**
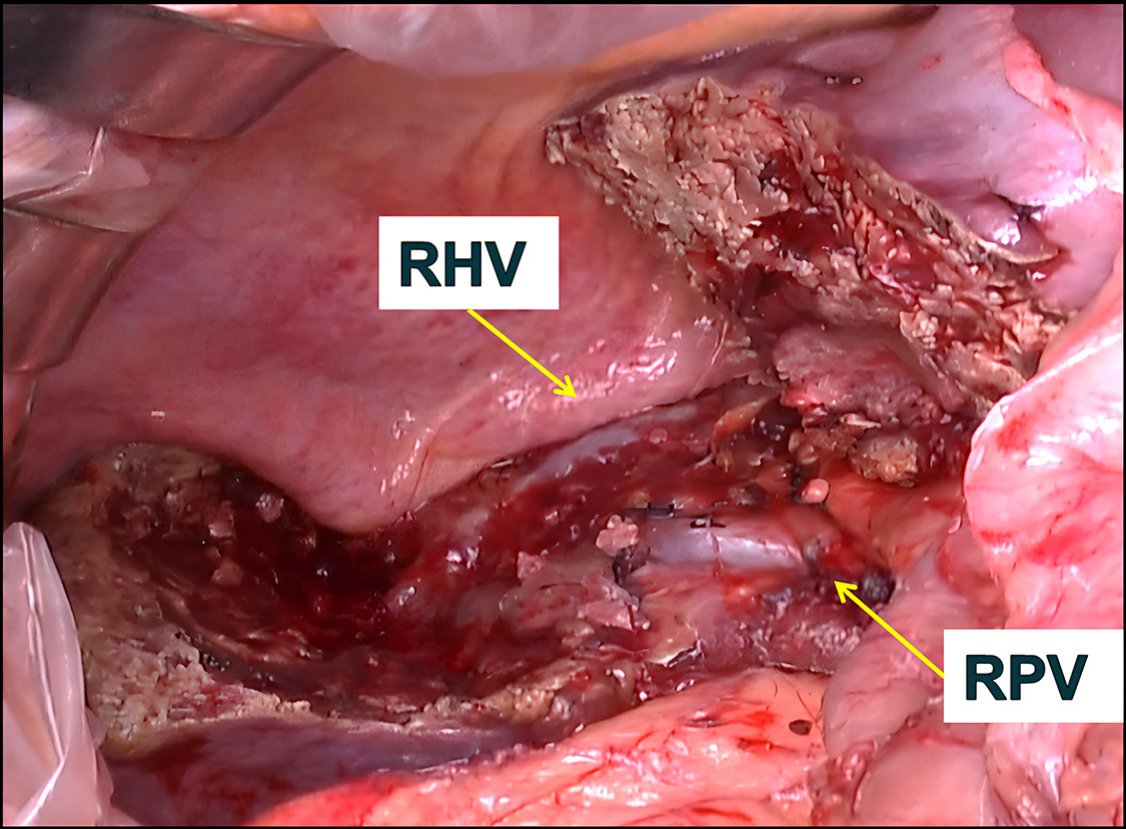
The resection surface following extended anterior sectionectomy (H4a + S5 + S8 resection), showing the RHV and RPV. RHV, right hepatic vein; RPV, right portal vein

We diagnosed the gallbladder cancer as cT3a (SI) (Hinf), cN0, cM0, cStage IIIA (Classification for the Management of Biliary Tract Cancer, 3rd Edition).^4)^ We performed an extended anterior sectionectomy (H4a + S5 + S8 resection), cholecystectomy, and lymphadenectomy of the hepatoduodenal ligament. During the separation of the boundary between the anterior and posterior sections, part of the tumor protruded, requiring a curved resection. The Glissonian pedicle from the posterior sectoral portal vein to the tumor (Glissonian pedicle of segment 6) was transected using the extrahepatic Glissonian approach, as was the Glissonian pedicle of the anterior section. The resected liver surface is shown (**Fig. 4**). The operation time was 10 h and 34 min, with a blood loss of 1480 mL. The postoperative bile leakage was managed conservatively. The patient was discharged home on postoperative day 35.

Pathological examination revealed 2 distinct tumors with different colors and relatively clear boundaries, contiguous at hepatic segment 8. Hematoxylin and eosin (HE) staining revealed 2 different lesions.

1. The hepatic lesion formed nests of varying sizes with a medullary growth pattern.
2. The gallbladder lesion displayed features of adenosquamous carcinoma with partial stratified differentiation and keratinization, as well as areas of sarcomatoid morphology with atypical spindle cell proliferation against a mucinous stroma.

**Fig. 5:**
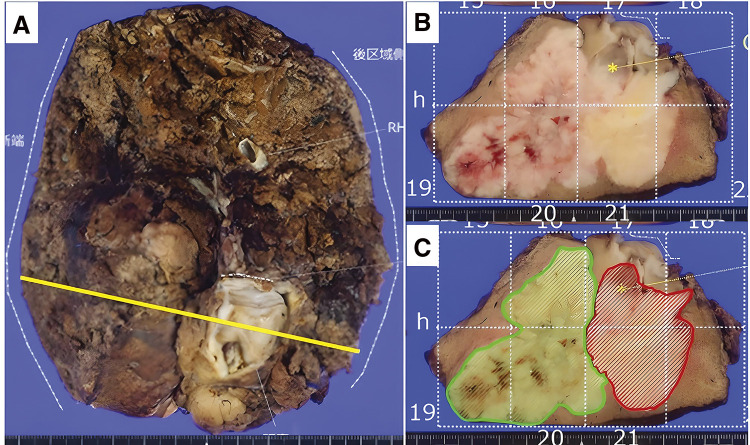
Resected specimen and maximum cut (yellow line) (**A**). Cut surface (**B**). Cut surface with mapping (**C**). The green area represents intrahepatic cholangiocarcinoma, and the red area represents carcinosarcoma.

**Fig. 6:**
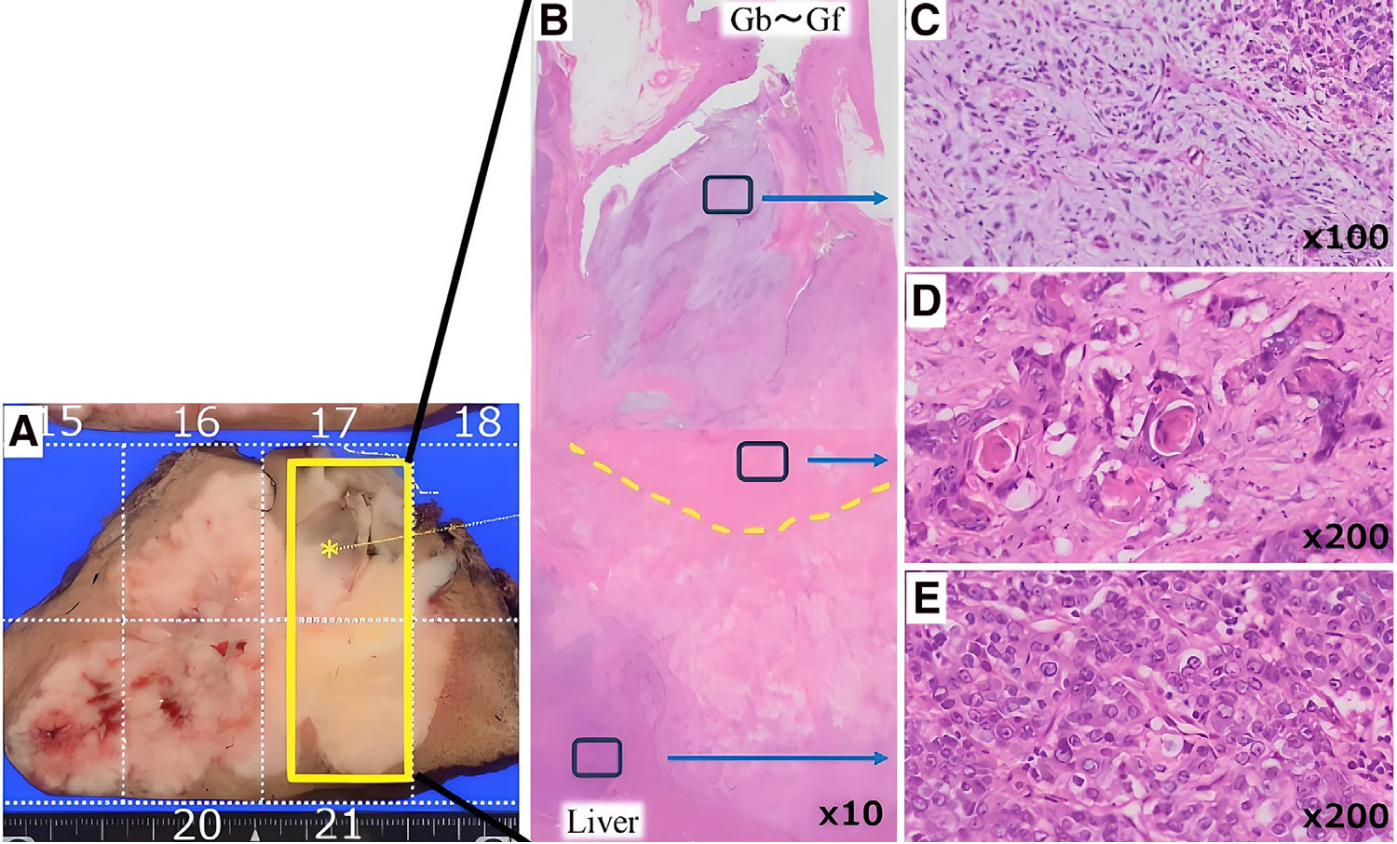
Macroscopic and histopathological findings (**A**). The yellow dotted line indicates the gallbladder bed (**B**). The gallbladder contains components resembling carcinosarcoma (**C**) and adenosquamous carcinoma, which are observed in the gallbladder wall and the liver (**D**). Poorly differentiated adenocarcinoma is observed to the left (**E**).

Normal hepatocytes were interspersed between the 2 lesions, with some areas invading each other. The gallbladder lesion had invaded the omentum (**Figs. 5** and **6**).

**Table 1** shows the immunohistochemical results.

1. In the hepatic lesion, hepatocyte-1 results were negative, glypican-3 and CK7 were partially positive, and CD56 was positive.
2. In the gallbladder lesion, the p40, CEA, and AE1/AE3 results were positive for the carcinoma component and negative for the sarcoma component.

**Table 1:** Immunohistochemistry results

	Hepatocyte-1	Glypican-3	CK7	CD56	p40	CEA	AE1/AE3
Liver							
ICC	−	+	+	+	–	–	+
Gallbladder							
ASC	−	−	−	−	+	+	+
Sarcoma	−	−	−	−	−	−	−

ICC, intrahepatic cholangiocarcinoma; ASC, adenosquamous carcinoma

Based on these findings, the patient was diagnosed with a collision carcinoma involving intrahepatic cholangiocarcinoma (pT3, pN0, cM0, and pStage III) and gallbladder carcinosarcoma (pT3a (SI), pN0, cM0, and pStage IIIA). Given the patient’s advanced age and the lack of evidence supporting chemotherapy for gallbladder carcinosarcoma, postoperative adjuvant chemotherapy was not initially recommended. However, it was ultimately administered at the patient’s strong insistence. S-1 was administered as adjuvant chemotherapy for only 1 week because of side effects. The patient survived without recurrence for 28 months.

## DISCUSSION

Collision carcinoma is a multiple primary carcinoma, defined as 2 independently arising tumors that come into contact with or partially invade each other.^5)^ Spagnolo et al.^3)^ emphasized the following diagnostic criteria for collision carcinoma: (1) the distribution of the 2 distinct histological types is clearly distinguishable; (2) each histological type is identifiable even at adjacent sites; and (3) the 2 components may intermingle at the collision site or exhibit a transitional zone where both components coexist. This is distinct from composite carcinoma, which is characterized by a continuous and densely intermixed histology.^6)^

In our case, macroscopic findings revealed 2 contiguous tumors with relatively clear boundaries and different colors in the hepatic segment 8. HE staining showed normal hepatocytes interspersed between the 2 lesions in some areas, and different tumor cells exhibited invasive tendencies toward each other in other areas. These findings satisfied Spagnolo’s diagnostic criteria.^3)^ confirming a diagnosis of collision carcinoma.

Collision carcinomas have been reported in various organs; however, reports in the liver and gallbladder are rare.^7)^ Fewer than 50 cases of collision carcinoma of the liver were found in PubMed between 1980 and 2024, with the majority involving hepatic neuroendocrine tumors and hepatocellular carcinoma.^8–12)^ Millien et al.^12)^ reported a case of collision carcinoma of the gallbladder, which is the only known case in this organ. This extremely rare case involved a unique combination of signet-ring cell carcinoma and large-cell neuroendocrine carcinoma.

This case could also be diagnosed from different perspectives. This case could be diagnosed as liver invasion by gallbladder carcinosarcoma. The reasons for this diagnosis include the following: (1) the overall macroscopic appearance was consistent with the typical presentation of liver invasion by gallbladder carcinoma, and (2) tumors with carcinosarcomatous components can exhibit diverse differentiation and phenotypes, leading to unexpected transitional appearances and progression patterns. Gallbladder carcinoma can invade the liver without distant metastasis^13)^ and display various pathological forms.^14)^ More than 100 cases of gallbladder carcinosarcoma have been reported, and these reports showed no specific findings on imaging examinations such as CT or MRI.^15)^ In general, the characteristic features of carcinosarcoma include large tumor size and heterogeneous enhancement with internal low-density areas on contrast-enhanced imaging.^16)^ In this case, the large tumor size and enhancement pattern were consistent with those reported previously, suggesting the typical morphology of carcinosarcoma. Therefore, the possibility of liver invasion by gallbladder carcinoma cannot be entirely ruled out. However, the pathological findings met the diagnostic criteria for collision tumors, and the 2 lesions did not clearly indicate a transition between the 2 components. Moreover, immunohistochemical staining suggested that these 2 lesions had different pathological features, and we ultimately diagnosed them as a collision tumor.

## CONCLUSIONS

Here, we report a rare case of collision carcinoma involving gallbladder carcinosarcoma (with an epithelial component of adenosquamous carcinoma) and intrahepatic cholangiocarcinoma. This is the first reported case of a collision tumor involving a gallbladder carcinosarcoma, and further case studies are warranted.

## ACKNOWLEDGMENTS

We would like to thank Editage (www.editage.jp) for the English language editing.

## DECLARATIONS

### Funding

Not applicable.

### Authors’ contributions

All authors contributed to the study conception and design.

### Availability of data and materials

Not Applicable.

### Ethics approval and informed consent

Ethical approval was not required, and informed consent was obtained from the patient.

### Consent for publication

Consent for publication has been obtained from the patient.

### Competing interests

The authors declare that they have no competing interests.
